# Book and Pamphlet Notices

**Published:** 1859-11

**Authors:** 


### BOOK AND PAMPHLET NOTICES.

PHYSICIANS' HAND BOOK OF PRACTICE. 1860. From S. C. Griggs & Co., Lake Street.

This little work is a pocket manual—if such an expression be correct,—and contains all that a physician may need for his memoranda, notes, accounts, etc. It is an improvement on anything of the kind which has fallen under our notice.

An Introduction to Practical Pharmacy. Designed as a Text Book for the Student, and as a Guide for the Physician and Pharmaceutist; with many Formulas and Prescriptions. By Edward Parrish, Principal of the School of Pharmacy, Philadelphia, etc. Philadelphia: Blanchard & Lea. P. 720. From W. B. Keen, Lake Street.

This is the second edition of a work which is already a standard on the subjects of which it treats. This work is not intended to supersede the National Pharmacopœia, but as a necessary supplement. The processes of preparing and dispensing medicine are fully detailed in the 2nd and 5th parts. Directions are given for the preparation of all the new medicines in use; and it appears to contain all the directions necessary to an accomplished pharmaceutist, who has already a sufficient knowledge of chemistry and materia medica. We recommend all physicians who are under the necessity of compounding their own medicines, and all apothecaries, to give it an examination.

---

The Action of Medicines in the System; or, "On the Mode in which Therapeutic Agents introduced into the Stomach produce their peculiar effects on the Animal Economy." By Frederick William Headland, M.D., B.A., F. L.S., Licentiate of the Royal College of Physicians, etc. etc. Third edition, revised and enlarged.

"On no question, perhaps, have scientific men differed more than on the theory of the action of medicines." Such is the opinion not only of our author but of all connected with medical pursuits. The book now before us, eminently calculated as it is to confer benefit on the science and practice of therapeutics, has already done much to lessen this difference of opinion, by the promulgation of correct principles. It has reached a third edition, which includes some matters not contained in those that preceded it. The arrangement, classification, and strictly logical method adopted; the conclusiveness and simplicity of the experiments in support of views, clear and philosophical; and the accuracy of expression with which these are communicated, —cannot but command attention and excite interest. The arrangement of the heads of the subjects in a number of distinct propositions, dividing each of these into minor propositions, which, taken together, imply the original one, is a method that will not fail to enable the student, by almost imperceptible steps, to keep pace with the arguments, and ultimately to share the convictions, of the author. Occasionally, he indulges in speculations and hypotheses, but not without having previously warned the reader that he was about to wander from the high-road of ascertained truth, over the ill-defined and indistinct by-paths of conjecture.

In the first chapter, devoted to introductory remarks, he sets forth the great importance and extent of the subject; shows in what manner he proposes to attain clearness and correctness of language and argument in the treatment of the several topics he is about to consider; and explains the scheme and arrangement which he means to follow in the execution of his design.

The second chapter contains a brief review of the opinions of other writers,—gives their classifications as "the best key to the main opinions" of their respective authors, and occasionally (and we must add, successfully) opposes doctrines hitherto unquestioned.

In the third chapter, he states his own opinions as to the *modus operandi* of medicines, and treats of their general modes in ten propositions. These we give in his own words:

PROP. I.

That the great majority of medicines must obtain entry into the blood, or internal fluids of the body, before their action can be manifested.

PROP. II.

That the great majority of medicines are capable of solution in the gastric or intestinal secretions, and pass without material change, by a process of absorption, through the coats of the stomach and intestines, to enter the capillaries of the Portal system of veins.

PROP. III.

That those medicines which are completely insoluble in water, and in the gastric and intestinal juices, cannot gain entrance into the circulation.

PROP. IV.

That some few remedial agents act locally on the mucous surface, either before absorption, or without being absorbed at all. That they are chiefly as follows:

*a.* Irritant Emetics.
*b.* Irritant Cathartics.
*c.* Superficial Stimulants, Sedatives, Astringents.

PROP. V.

That the medicine, when in the blood, must permeate the mass of the circulation, so far as may be required to reach the parts on which it tends to act.

That there are two possible exceptions to this rule:

*a.* The production of sensation or pain at a distant point.

*b.* The production of muscular contraction at a distant point.

PROP. VI.

That while in the blood the medicine may undergo changes, which in some cases may, in others may not, affect its influence. That these changes may be—

*a.* Of Combination.

*b.* Of Reconstruction.

*c.* Of Decomposition.

PROP. VII.

That the first class of medicines, called Hæmatics, act while in the blood, which they influence. That their action is permanent.

1. That of these some, called Restoratives, act by supplying, or causing to be supplied, a material wanting; and may remain in the blood.
2. That others, called Catalytics, act so as to counteract a morbid material or process; and must pass out of the body.

PROP. VIII.

That a second class of medicines, called Neurotics, act by passing from the blood to the nerves or nerve-centres, which they influence. That they are transitory in action.

1. That of these some, called Stimulants, act so as to exalt nervous force, in general or in particular.
2. That others, called Narcotics, act so as first to exalt nervous force, and then depress it, and have also special influence on the intellectual part of the brain.
3. That others again, called Sedatives, act so as to depress nervous force, in general or in particular.

PROP. IX.

That a third class of medicines, called Astringents, act by passing from the blood to muscular fibre, which they excite to contraction.

PROP. X.

That a fourth class of medicines, called Eliminatives, act by passing out of the blood through the glands, which they excite to the performance of their functions.

The chief objects for which he designed the fourth chapter are, as he tells us, "to illustrate some general principles of the action of medicines which have been laid down in the propositions, and show in what manner they are applicable to special cases, and to enter into certain details respecting the more important remedies."

We thus see his classification embraces four great groups of medicines, to each of which one of the last four propositions refers. The first group he divides into medicines restoring to the blood a substance natural to this fluid, and not necessarily excreted from it; and into such as are not its natural constituents, and therefore such as sooner or later must be excreted from it. The applicability of the former depends on the absence of a natural substance; that of the latter, on the presence of an unnatural agency. The second group includes nearly all the powerful poisons that act after passage into the blood, and whose peculiar mode of action is in many respects involved in obscurity. The third, less important and less obscure in action, seem to owe their remedial properties both to chemical and vital influences. The fourth form an extensive class, and though their efficacy in the removal of disease, and, to some extent, the manner in which this is exerted, were not unknown to our predecessors, yet the precise method by which, in those instances, causes proved adequate to the production of corresponding effects, was a problem reserved for solution by more recent observers.

Some years ago the opinions entertained by the profession as to the mode of action of medicines might be referred to three different heads. Some maintained medicines were absorbed by an order of vessels that discharged this particular office of absorption, and this only; others, that absorption was effected by the venous system; and others still, that though the action of some substances might be the result of absorption, yet this was not necessary to the development of the action of all; as many operated through the sentient extremities of the nerves. That the sentient extremities of nerves are the media between medical agents and the brain is a doctrine opposed by the author. We are not prepared altogether to ignore the possession of some property of this kind to the nervous system. We confessedly share, in this respect, the guilt of medical heresy that attaches to some of the most competent observers in the profession. Amongst others, Sir B. Brodie thinks that the action of a medicine or a poison may be partly transmitted through the nervous system. We are aware of there being arguments advanced against this opinion—of which, perhaps, not the least forcible is, that it is unsupported by analogical reasoning—that, in fact, each fibril of a nerve has some particular office assigned to it, and that there is no instance in the animal economy of a nervous filament being capable of performing a double office. But in questions of science we must bear in mind that analogy is not proof; and a principle should be proved before analogy can be available in giving weight to evidence. We will not, however, dwell further on this matter, but proceed with the main subject.

Dr. Headland states that the great majority of medicines are absorbed through the coats of the stomach and intestines, and pass through to the portal veins—that continuity of nerve is not necessary—that the circulation of the blood is sufficiently quick to account for the operation of even those poisons which act most rapidly by influencing the nerve centres, and that the great majority of medicines have been detected in the blood, and found in the secretions formed out of it. Some facts ascertained of late years and now generally received, serve as the foundation of these several propositions. It is conceded that the secretion of the stomach is acid,—that of the liver and pancreas, alkaline; and that these secretions act on different principles taken as food or medicine. In the stomach, the venous system is the agent of transmission of absorbed matter; in the intestines, not only the venous system, but also the lacteal vessels perform this office. Fatty matters, whether food or medicine, are not supposed to pass directly into the blood through the mesenteric and portal veins, but are carried along the lacteal system, after having been emulsified and saponified by the bile and pancreatic secretion. Under this head we must include resins, and medicines owing their efficacy to resin. By the action of the lactic acid and pepsine of the gastric juice, nitrogenous and other nutritive matters are dissolved and absorbed by the portal venous system.

From the researches of experimental physiologists, it would seem that starch and substances referable to this principle are alike indebted to these different agents for their successive transformation into dextrine and glucose, until ultimately they result in combustion and the formation of carbonic acid. Dr. Headland does not think the lacteals are ever engaged in the absorption of medicinal solution, with the exception, perhaps, of fats and fixed oils, for the following reasons:

In the first place, it appears from the researches of Bernard and others that the lacteal system is a special arrangement for the absorption of fatty substances, and that other matters, such as albuminous compounds, pass for the most part into the veins, and thence to the liver. Besides, it seems that these lacteal absorbents are only in action during the digestion of food, when the epithelium on the surface of each villus becomes loosened, in order to allow to the chyle an easier access to the lacteal within it. So that it is likely that a small portion of the fluid or soluble substance would be insufficient to rouse them to action. And, in the third place, direct experiments of a decisive kind have been made on this point. Magendie has found that the ligature of the lacteal trunks does not prevent the occurrence of poisoning from agents introduced into the bowels. And Tiedemann and Gmelin have carefully sought in the chyle for a number of different medicines administered to animals in their food, and have been unable to detect any of them there. So that, with the exception, perhaps, of fats and fixed oils, we may reasonably conclude that no medicinal substances pass into the system through the lacteals, but that all are absorbed by the veins or capillary vessels.

Two distinct classes of substances are already stated to be susceptible of absorption. Besides these, there are mineral substances soluble in water, in acids, or in alkalies; and vegetable products soluble in water. All minerals soluble in water, we are told, are, without exception, absorbed in the stomach and intestines. It has been argued by some that the purgative effect of saline purgatives of greater specific gravity than serum is in obedience to the physical law,—that the lighter of two fluids of various density passes through to the heavier;—that, therefore, when a saline is so diluted as to be lighter than serum, it is absorbed, and, when absorbed, produces diuresis; but that when its specific gravity is greater than serum, it purges, by causing a passage of the serum outwards into the intestines, thas producing a watery evacuation.

This theory is opposed by Dr. Headland. He believes that both solutions are absorbed, and the effect, whether purgative or diuretic, mainly depends on the quantity of the salt, as the kidneys will not eliminate beyond a certain amount of it. In support of his views, he made the following experiments:

EXP. 1—A sufficient quantity of white sugar was dissolved in four ounces of water to raise its specific gravity to 1·028, that of the serum of the blood. A sufficient quantity of Sulphate of Magnesia was dissolved in another ounce of water, to render it of the density of 1·060. This heavier solution was introduced into an open wide tube, closed completely below by a clean piece of bladder. It was introduced into a small vessel containing the solution of sugar, and arranged so that the height of the two liquids should correspond. After standing for some time, the inner solution measured two drachms more, and the specific gravity had sunk to 1·050. The outer solution, after making up exactly the loss by evaporation, was found to have risen in density to 1·040. On adding a small quantity of each of the solutions of Phosphate of Soda and Carbonate of Ammonia, a copious precipitate took place, indicating the presence of magnesia. Thus it appeared that the fluids passed both ways, some of the heavy solution of magnesia finding its way through to the lighter solution of sugar. The tendency of this process was evidently to an equalization of their densities, both by endosmose one way, and by exosmose the other. Thus, apparently, would it be with a saline purgative, and with the serum of the blood.

EXP. 2.—Three drachms of Sulphate of Magnesia (a very mild purgative dose) were dissolved in ten ounces of water, and thus administered to a healthy young man. It produced, after some time, slight purging, and some diuresis. The urine, when tested, contained only a very little more than the usual quantity of magnesia. The quantity in the dose was less than five per cent. of the solution, and thus, according to the endosmotic theory, should have produced no purging.

EXP. 3.—Six drachms of the same salt were given in eighteen ounces of water to the same patient. After a few hours it caused very copious and long-continued watery purging. The urine did not seem to be increased, and contained no excess of magnesia. It seemed, that in spite of the dilution, the quantity of the salt was so large, that it could not pass off by the kidneys, and so was eliminated from the blood by the bowels, in the same way as other purgative medicines. (*Vide* Cathartics, and Chap. IV.)

Exp. 4.—This trial was the reverse of the last. Two scruples of Acetate of Potash were dissolved in three drachms of water, and thus administered. The solution then contained about twenty per cent. of the salt. According to the endosmotic theory, it should have caused only slight purging, on account of its density. It did not do so, but produced diuresis. The dose was so small, that after absorption it was easily eliminated by the kidneys. The alkalinity of the urine which resulted was a proof of the absorption of the salt, and its passage into this secretion in the form of a carbonate. (See Prop. VI.)

Exp. 5.—(In Chap. IV., Art. Sulphate of Magnesia, will be detailed some experiments designed for the purpose of still further demonstrating the *modus operandi* of this salt and others like it. One of these may be quoted here, as bearing directly upon the subject in hand. It proves that a dense solution of Sulphate of Magnesia is absorbed in the intestinal canal.)

Three drachms of Sulphate of Magnesia, dissolved in three ounces of water, forming a solution having the density of 1·060, were injected down the throat of a healthy dog. It was killed after three-quarters of an hour, and the whole contents of the stomach and intestines, none of which had been lost either by vomiting or purging, were subjected to a careful analysis. The result showed that only fifty-five grains of the salt remained out of the three drachms. The rest must have been absorbed into the blood.

To sum up these experiments. The first shows how, in an experiment made out of the body, a dense fluid will pass through to a light one on the opposite side of a membrane, the densities of the two being at the same time gradually equalized. The second and third demonstrate that a solution of low specific gravity will occasion purging, whereas, according to the *Endosmotic theory*, it should not do so. And the fourth and fifth show that a dense solution is absorbed; whereas, according to the *Endosmotic theory*, it should not be absorbed.

We believe there are few at present who will deny that some diseases originate in the blood, and that the most rational mode of treatment must be in meeting the evil in its stronghold—*in fonte* and *originé*. We know we are, in many instances, now possessed of the means of successfully dealing with disease, whether this depends immediately on the absence of some material in the blood necessary to health or on the presence of what may be positively prejudicial to it. These means consist in what Dr. Headland terms Hæmatics. He divides them into Restoratives and Catalytics, as they are respectively adapted to the two conditions above stated. This classification especially recommends itself by its simplicity. There is an air of truth about it, that unavoidably leads a reader, conversant with disease, to the conviction that it rests on no frail foundation, but that it is destined to remain one of the established facts of science. "Each Restorative has in healthy blood a substance analogous to or identical with itself: it replaces this when deficient. Not so with other Hæmatics. There is, in general, nothing in the blood corresponding to them."

Dr. Headland remarks that Catalytic medicines sometimes simulate the action of particular diseases, and that the disciples of Hahnemann fancy they derive from this a sanction to their absurd theory. The following explanation suggested by our author is ingenious: "This partial resemblance is probably due to the fact that both disease and remedy produce a series of changes in the same set of particles in the blood. If it were not so, the remedy could not meet the disease. It would be out of its province, as not acting at all in the same sphere. But that the actions are essentially different is sufficiently proved by the fact that they counteract each other. The remedy moreover is often of equal efficacy in other different disorders."

We now pass on to a review of the eighth proposition; and here, from the obscurity in which nature veils her proceedings, we are deprived of much of the interest we felt in the consideration of the former proposition. There we had the light of Chemistry to elucidate many things that in former days could be but guessed at;—in the part of the subject we are now come to, we can only grope in the dark. Here the aid of chemical science is wholly unavailing, and those influences which we vaguely term vital, seem to reign with undisputed sway over the many incomprehensible phenomena of the Nervous System. The author, however, is not satisfied without venturing a theory to account for the action of the class of medicines called Neurotics. We insert his own language. "It is generally believed, among scientific men, that each particle of a compound body is made up of a number of indivisible atoms, each of which is inconceivably minute in size. And as these compound bodies have each a peculiar chemical constitution, so must each of their ultimate parts be composed of a peculiar arrangement of simpler atoms, and thus have a certain shape of its own, more or less different from the shape of every other compound atom. Both the substance of a nerve, and the active part of a nerve-medicine, consist of a number of definite compound atoms. And it is possible that the atom of a stimulant medicine may be of such a shape as that it shall be unable to coincide with, or to fit into, the series of atoms forming the sensitive surface of the nerve, and thus irritate this when brought into contact with it; and that the compound atoms of a sedative may so arrange with these nerve particles as to fit among and extinguish the salient points, and annihilate their natural sensibility."

This is of course purely conjectural, and its truth or falsehood must, from our finite powers of observation, be wholly insusceptible of proof. It is not, however, probable, from analogical reasoning, that nature accomplishes her work in a living body by means so mechanical. Such notions are more suited to the days of corpuscular pathology—the days of angles, spherules and frictions derived from the Cartesian philosophy—than in these of advanced development in science. They cannot, therefore, receive a serious and formal refutation. We have neither confidence in this theory of the action of Neurotic medicines by atomic shape nor in another of the chemical school, though sanctioned by Liebig, Snow and others. As this latter, however, has not received Dr. Headland's sanction, we will not at present consider it further.

We must now bring our notice to a close, having already exceeded the space we originally intended to assign it.

This is a book, the intrinsic merit of which reveals itself in proportion to the pains expended in working the deep mine of intellectual wealth with which it is enriched. The ore, however, throughout, is not of equal value. What is contained under the seventh proposition defies censure, and is beyond our feeble commendation. The fourth chapter, devoted to "the action of the more important medicines in particular," neither indicates the research nor the matured mind displayed in the preceding chapters. These are bestowed in unequal proportions, through its various parts. This alone would constitute a blemish; but, in addition, there is an inaccuracy of language that takes off from the seemliness of its appearance. These are blemishes which can be easily rectified, and that would now have most probably escaped observation, had not the book by its very excellence invited animadversion; for in proportion to the worth or purity of a thing, so by contrast is any defect of it more easily discerned. Yet with all the trifling imperfections that even the most fastidious criticism can expose, this work must have an undisputed title to pre-eminence in this department of medical literature.

---

We are in receipt of the following works from W. B. Keen:

Pathological and Practical Observations on Diseases of the Alimentary Canal, Œsophagus, Stomach, Cæcum, and Intestines. By S. O. Habershon, M.D., Londin., Fellow of the Royal College of Physicians; Assistant Physician to Guy's Hospital, and Lecturer on Materia Medica and Therapeutics; late Demonstrator of Morbid Anatomy, and Curator of the Museum at Guy's, etc. etc. Philadelphia: Blanchard & Lea. 1859.

A Practical Treatise on the Diagnosis, Pathology, and Treatment of Diseases of the Heart. By Austin Flint, M.D., Professor of Clinical Medicine, etc., in the New Orleans School of Medicine; Visiting Physician to the New Orleans Charity Hospital; Honorary Member of the Medical Society of Virginia, of the Kentucky State Medical Society, of the Medical Society of Rhode Island, of the Pathological Society of Philadelphia, etc. Philadelphia: Blanchard & Lea. 1859.

Also, from Jones & White, Dental Depot, Chicago:

A System of Dental Surgery. By John Tomes, F.R.S., Dentist to the Dental Hospital of London, and to the Middlesex Hospital. With Two Hundred and Seven Illustrations. Philadelphia: Lindsay & Blakiston. 1859.

A Practical Treatise on Operative Chemistry. By J. Taft, Professor of Operative Dentistry in the Ohio College of Dental Surgery. With Eighty Illustrations. Philadelphia: Lindsay & Blakiston. 1859.

